# A combined DEAV-BWM approach for effective evaluation and ranking of biomass materials in charcoal briquette production

**DOI:** 10.1016/j.mex.2024.103075

**Published:** 2024-11-28

**Authors:** Narong Wichapa, Pariwat Nasawat, Nattapat Kanchanaruangrong, Atchara Choompol

**Affiliations:** a Department of Industrial Engineering, Faculty of Engineering and Industrial Technology, Kalasin University, Kalasin 46000, Thailand; b Department of Logistics and Process Engineering, Faculty of Industrial Technology, Rajabhat Rajanagarindra University, 24000, Thailand; c Department of Industrial Management Engineering, Faculty of Industrial Technology, Rajabhat Rajanagarindra University, 24000, Thailand; d Department of Computer Engineering, Faculty of Engineering and Industrial Technology, Kalasin University, Kalasin 46000, Thailand

**Keywords:** Data envelopment analysis variant, Best-worst method, Biomass, Charcoal briquette, Multi-attribute decision making, Data envelopment analysis, Method of integrating a novel Data Envelopment Analysis Variant (DEAV) model, and Best-Worst Method (BWM) for assessing the potential of biomass materials in charcoal briquette production

## Abstract

The utilization of agricultural waste for producing charcoal briquettes is gaining significant attention as a sustainable alternative energy source. Converting these residues into charcoal briquettes not only addresses energy shortages but also provides an efficient solution for managing agricultural waste, contributing to environmental sustainability. This study proposes a novel methodology integrating a Data Envelopment Analysis Variant (DEAV) with the Best-Worst Method (BWM) to assess and rank biomass materials for charcoal briquette production. The DEAV-BWM model enhances the evaluation process by considering multiple criteria simultaneously and incorporating both efficiency and consensus among different evaluation methods. The key highlights of the methodology are as follows:

• A novel method, called the DEAV model, for evaluating efficiency scores has been established, incorporating both Data Envelopment Analysis (DEA) and Multi-Attribute Decision Making (MADM) concepts.
• This paper introduces a novel hybrid method that combines the DEAV model with the BWM to evaluate and rank biomass materials for charcoal briquette production, enhancing the reliability and robustness of the decision-making process.
• The proposed method can be adapted and applied to other areas of biomass utilization and beyond, providing a versatile tool for researchers and practitioners in the field of sustainable energy, engineering, and operations research.

## Graphical abstract

**Figure:**
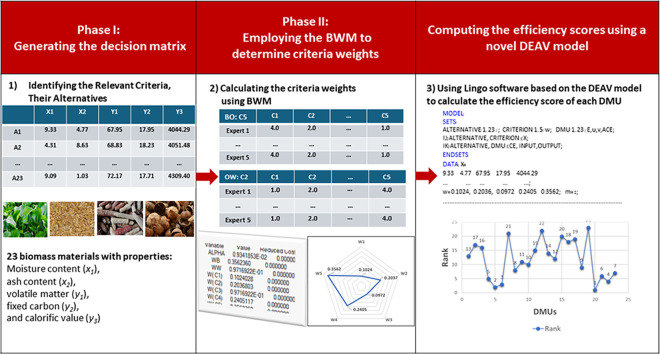

Specifications table

**Table:** 

Subject area:	Engineering
More specific subject area:	Assessing the Potential of Biomass in Briquette Production
Name of your method:	Method of integrating a novel Data Envelopment Analysis Variant (DEAV) model, and Best-Worst Method (BWM) for assessing the potential of biomass materials in charcoal briquette production
Name and reference of original method:	“None”.
Resource availability:	Not applicable.

## Background

The concept of utilizing biomass materials for producing charcoal briquettes is attracting significant attention as a sustainable alternative energy source. Biomass, derived from organic materials such as agricultural waste (e.g., rice straw, corn cobs, and coconut shells), is abundant yet underutilized in many agricultural nations, particularly Thailand. The production of charcoal briquettes from biomass not only mitigates energy shortages but also offers an efficient solution for managing agricultural waste [[1], [2], [3]]. Biomass-based charcoal briquettes provide several advantages, including high energy efficiency, reduced environmental impact, and the potential to alleviate rural energy poverty by offering a reliable source of cooking and heating fuel [4,5]. The production process of charcoal briquettes involves several critical steps and necessitates specific properties of the biomass materials to ensure high-quality output. Essential properties include low moisture content, low ash content, high calorific value, and high fixed carbon content [[6], [7], [8], [9], [10]].

Selecting suitable biomass materials for briquette production poses a Multiple Attribute Decision Making (MADM) challenge due to the need to evaluate various properties of biomass simultaneously. Several tools and methods have been developed to address this MADM challenge, including the Technique for Order of Preference by Similarity to Ideal Solution (TOPSIS) [11], Weighted Aggregates Sum Product Assessment (WASPAS) [12], Additive Ratio Assessment (ARAS) [13], Complex Proportional Assessment (COPRAS) [14], Multi-Objective Optimization by Ratio Analysis (MOORA) [15], and Grey Relational Analysis (GRA) [16]. These methods can be used for solving various MADM problems. Certainly, the challenges of evaluating and ranking biomass for briquette production can be addressed using these methods, which facilitate the ranking and selection of the best biomass materials based on multiple criteria, ensuring the efficient production of high-quality briquettes.

This paper contributes to the existing body of knowledge by proposing a novel methodology for evaluating and ranking biomass materials for charcoal briquette production. The proposed method integrates the Data Envelopment Analysis Variant (DEAV) model with the Best-Worst Method (BWM), enhancing the reliability of the evaluation process. The proposed method was tested on 23 biomass materials, demonstrating its effectiveness in identifying the most suitable materials for high-quality charcoal briquette production. The contributions of this paper are as follows.

1. This paper introduces a novel methodology that combines the Data Envelopment Analysis Variant (DEAV) with the Best-Worst Method (BWM) [17] for evaluating and ranking biomass materials, enhancing the reliability and robustness of the decision-making process.
2. The proposed framework provides a comprehensive approach to addressing MADM challenges in biomass selection, considering both efficiency and consensus among different evaluation methods.
3. The methodology was empirically tested on 23 different biomass materials, demonstrating its practical effectiveness in identifying the most suitable materials for high-quality charcoal briquette production.
4. By integrating DEAV with BWM, the proposed method improves the reliability of the evaluation process, particularly in scenarios involving multiple criteria and complex decision-making environments.
5. The methodology presented in this paper can be adapted and applied to other areas of biomass utilization and beyond, providing a versatile tool for researchers and practitioners in the field of sustainable energy, engineering and operations research.

## Method details

### Proposed method

**Fig. 1:**
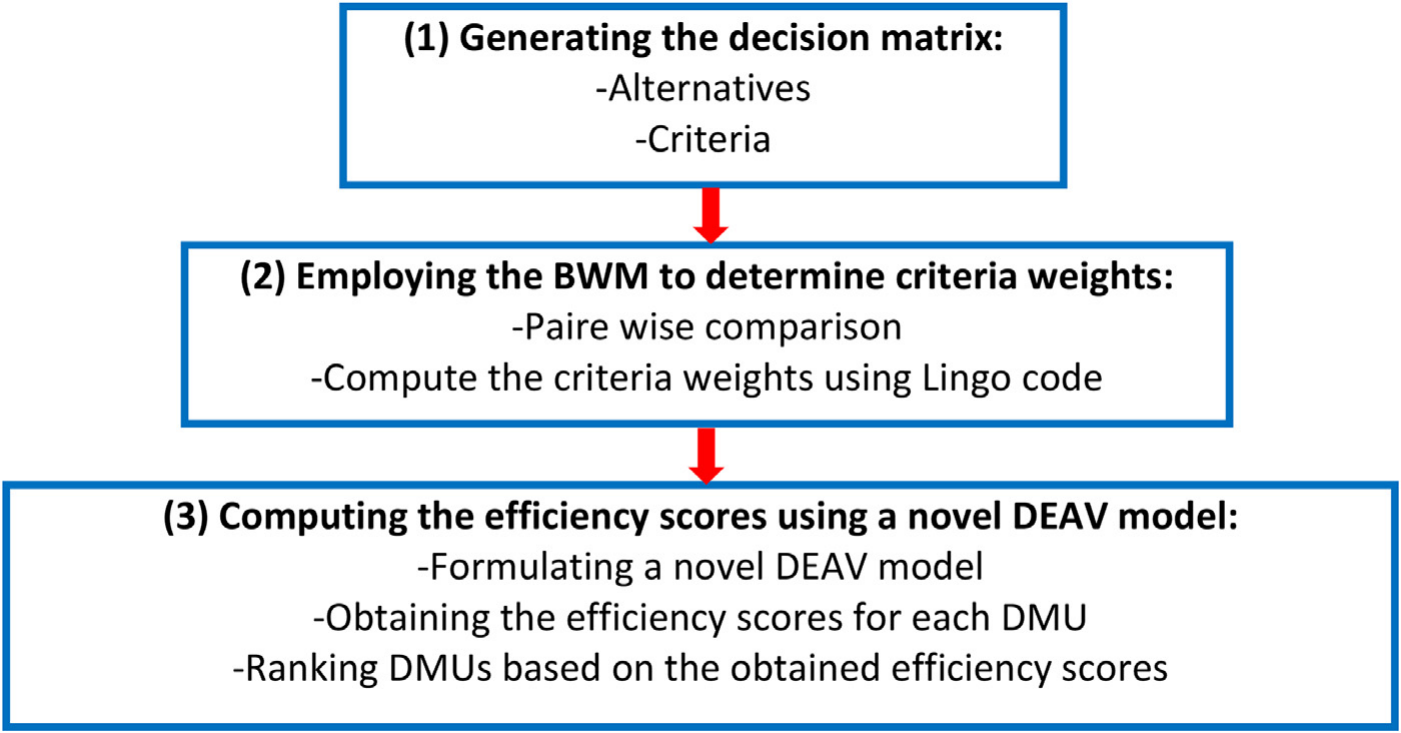
The proposed framework.

This paper comprises several essential steps: (1) generating the decision matrix, alternatives, and criteria; (2) employing the BWM to determine criteria weights; and (3) computing the efficiency scores using a novel DEAV model. The structure of this paper is depicted in Fig. 1.

(1) Generating the decision matrix

All biomass materials or alternatives, along with the corresponding relevant properties or decision criteria, will be systematically incorporated to construct the decision matrix. This approach ensures that each biomass material is thoroughly evaluated against all pertinent criteria. The methodology and construction of the decision matrix are elaborated in detail in Eq. (1), which serves as a foundational component of our analysis.

(1) $$\begin{array}{c} \;\;\;\;\;\;\;\;\;\;\;\;\;\;\;\;\;\;\;\begin{array}{cccc} C_{1} & \;\;C_{2} & \cdots & \;C_{m} \end{array}\\ X=\begin{array}{c} A_{1}\\ A_{2}\\ \vdots \\ A_{n} \end{array}\left[\begin{array}{cccc} x_{11} & x_{12} & \ldots & x_{1m}\\ x_{21} & x_{22} & \ldots & x_{2m}\\ \vdots & \vdots & \ldots & \vdots \\ x_{n1} & x_{n2} & \ldots & x_{nm} \end{array}\right] \end{array}$$

In Eq. (1), *A_k_* (where *k* = 1,2,…, *n*) represents the alternatives, *C_j _* (where *j* = 1,2,…,*m*) signifies the criteria related to the performance of these alternatives, and *x_kj_* represents the value of alternative *k* with respect to criterion *j*.

(2) Employing the BWM to determine criteria weights

The BWM [17] is widely acknowledged as a powerful and efficient approach for determining criteria weights through the pairwise comparison technique. This method stands out for its ability to minimize inconsistency in the decision-making process, thereby enhancing the reliability of the results. The BWM operates through a structured procedure that involves several key steps [18,19]:

**Step 1:** Define the criteria for evaluating alternatives as *C_1_,C_2_*,…,*C_m_*.
**Step 2:** Record comparisons between alternatives in the matrix cells.
**Step 3:** Select the alternative with the most favorable and least favorable performance for each criterion.
**Step 4:** Experts or decision-makers assign scores to the best criterion using a Likert scale from 1 to 9, comparing it with each criterion indexed as *a_Bj_*.
**Step 5:** Assign scores to the worst criterion using a Likert scale from 1 to 9, comparing it with each criterion indexed as *a_jw_*.
**Step 6:** Calculate the optimal weights for each criterion, along with the consistency ratio. The constructed model is then outlined.

**Objective function:**

(2) $$Z=\text{min}\;\varepsilon^{L}$$

**Subject to:**

(3) $$\left|w_{B}-a_{Bj}w_{j}\right|\leq \varepsilon^{L},\;\;\forall j$$

(4) $$\left|w_{j}-a_{jW}w_{W}\right|\leq \varepsilon^{L},\;\;\forall j$$

(5) $$\sum_{j=1}^{n}w_{j}=1$$

(6) $$w_{j}\geq 0,\;\;\forall \;j$$

In the mathematical model of BWM, the objective function presented in Eq. (2) seeks to minimize the degree of inconsistency within the pairwise comparisons. Eq. (3) ensures that the absolute difference between the weight of the best criterion and each criterion *j* is within the specified consistency index. Similarly, Eq. (4) ensures that the absolute difference between the weight of the worst criterion and each criterion *j* is also within the consistency index. Additionally, Eq. (5) guarantees that the sum of the weights for all criteria equals one, while Eq. (6) ensures that the weight of each criterion is non-negative. By solving Eqs. (2) to (6) using Lingo software, the optimal criterion weights and the consistency ratio can be determined. These criterion weights will then be incorporated into the DEAV model for ranking alternatives.

(3) Computing the efficiency scores using a novel DEAV model

Charnes, Cooper, and Rhodes [20] introduced the mathematical model of Data Envelopment Analysis (DEA), known as the CCR model. This mathematical model has been applied across various research fields, including engineering, economics, and others [[21], [22], [23], [24]]. The advantage of using the DEA (CCR model) for efficiency measurement is its ability to evaluate the performance of numerous production units, each with multiple factors, without the need for data normalization prior to calculation. Furthermore, it does not require the pre-specification of factor weights, as the DEA model can optimally determine the weight set for each production unit [25,26]. One limitation of the DEA model is its inability to rank all production units when multiple DMUs achieve an efficiency score of 1, indicating they are efficient [[27], [28], [29]]. To overcome this limitation, this research combines the DEA model with the MADM approach, which assigns weights to each criterion for a more refined efficiency assessment. This research develops the DEA and MADM concepts to overcome the limitations of traditional DEA in ranking all alternatives or production units. In this model, the importance weights of each criterion are considered. The research assigns these weights using the Best Worst Method (BWM). The DEAV model can be defined as follows:

**Step 1:** Formulating a novel DEAV model. Consider a set of Decision-Making Units (DMUs), denoted as DMU*_j_*, each defined by a collection of inputs *x_ij_* for *i* = 1,…,*m* and a collection of outputs *y_ri_* for *r* = 1,…,*s*. Let *u_k_* represent the weights assigned to the evaluated output *k* and *v_k_* represent the weights assigned to the evaluated input *k*. The DEAV model is an advanced approach designed to evaluate the performance of DMUs by incorporating multiple inputs and outputs. This research aims to transform the non-linear DEAV model into a linear programming model to enhance its computational efficiency and applicability. The formulation of the novel linear programming model for DEAV is presented below. Initially, the DEAV model is represented in a non-linear form, as shown in Eqs. (7) to (9).

**Objective function:**

(7) $$\max\theta_{k}=\left(\sum_{r=1}^{s}u_{k}\cdot y_{rk}^{w_{r}}\right)/\left(\sum_{i=1}^{m}v_{k}\cdot x_{ik}^{w_{i}}\right)\leq 1,$$

**Subject to:**

(8) $$\left(\sum_{r=1}^{s}u_{k}\cdot y_{rj}^{w_{r}}\right)/\left(\sum_{i=1}^{m}v_{k}\cdot x_{ij}^{w_{i}}\right)\leq 1,$$

(9) $$\;u_{k},v_{k}\geq 0.$$

where *w_i_* and *w_r_* are the weights for input and output variables, respectively. In this study, these weights are determined through BWM. Eqs. (7) to (9) represent a non-linear programming model. Eqs. (10) to (13) demonstrate the conversion of this model into a linear programming model.

**Objective function:**

(10) $$\theta_{k}=\max\left(\sum_{r=1}^{s}u_{k}\cdot y_{rk}^{w_{r}}\right),$$

**Subject to:**

(11) $$\left(\sum_{i=1}^{m}v_{k}\cdot x_{ik}^{w_{i}}\right)=1,$$

(12) $$\;\left(\sum_{r=1}^{s}u_{k}\cdot y_{rj}^{w_{r}}\right)-\left(\sum_{i=1}^{m}v_{k}\cdot x_{ij}^{w_{i}}\right)\leq \;0,\forall j$$

(13) $$\;u_{k},v_{k}\geq 0.$$

The specified equations are components of the DEAV model, a variant of DEA that utilizes linear programming to evaluate the efficiency of DMUs. The specifics of each equation can be elucidated as follows.

**Objective function (as delineated in**Eq. (10)**:**$\theta_{k}=\max\left(\sum_{r=1}^{s}u_{k}\cdot y_{rk}^{w_{r}}\right)$, the objective function aims to maximize $\theta_{k}$, the efficiency score of the *k*-th DMU. In this context:

• $\theta_{k}$ is the efficiency score for DMU *k*, which is the main target of maximization.
• *u_k_* represents the weight assigned to the output *r* for DMU *k*.
• $y_{rk}^{w_{r}}$ is the *r*-th output for DMU *k*, with an associated weight *w_r_*.

In essence, the objective function seeks to maximize the weighted sum of outputs for DMU *k*, thus achieving the highest efficiency possible for that DMU.

**Constraints:** The model is subject to the following constraints:

**1. Normalization Constraint (as delineated in Eq. (11):**

$(\sum_{i=1}^{m}v_{k}\cdot x_{ik}^{w_{i}})=1$, this constraint ensures that the weighted sum of inputs for DMU *k* is normalized to 1. Here:

• *v_k_* represents the weight assigned to the input *i* for DMU *k*.
• $x_{ik}^{w_{i}}$ is the *i* th input for DMU *k*, with an associated weight *w_i_*.

This constraint maintains a consistent basis for comparing efficiency across all DMUs by setting the sum of the weighted inputs to a fixed value of 1.

**2. Inequality Constraint for Efficiency (as delineated in Eq. (12):**

$\left(\sum_{r=1}^{s}u_{k}\cdot y_{rj}^{w_{r}}\right)-\left(\sum_{i=1}^{m}v_{k}\cdot x_{ij}^{w_{i}}\right)\leq \;0,\forall j$, this constraint implies that for each DMU *j*, the weighted sum of outputs should not exceed the weighted sum of inputs. This ensures that no DMU's efficiency exceeds a feasible range and allows a fair comparison among all DMUs.

**3. Non-negativity Constraint (as delineated in Eq. (13):**

$u_{k},v_{k}\geq 0$, This constraint requires that all weights (*u_k _* and *v_k_*) are non-negative, as negative weights would not have a meaningful interpretation in this context.

**Step 2:** Obtaining the efficiency scores for each DMU. The model specified in Eq. (8) will be coded in Lingo to calculate the efficiency scores for evaluating the alternatives.
**Step 3:** Ranking DMUs based on the obtained efficiency scores. Once the efficiency scores for each alternative or decision-making unit (DMU) have been obtained, the DMUs will be ranked in descending order based on their efficiency scores.

### Numerical example

The concept of converting biomass into briquettes is a promising solution for addressing energy shortages in agricultural countries. It effectively transforms agricultural waste into valuable products. However, optimizing biomass for briquette production requires assessing the potential of various biomass types. This assessment must consider key fuel properties, as multiple factors influence biomass fuel efficiency, making the evaluation of each type challenging.

This research introduces the DEAV-BWM method to measure biomass efficiency by identifying key fuel properties such as moisture content (*x_1_*), ash content (*x_2_*), volatile matter (*x_3_*), fixed carbon (*y_1_*), and calorific value (*y_2_*). Moisture content, ash content and volatile matter are considered input factors (*x*_i_), while fixed carbon, and calorific value are output factors (*y_r_*). Based on the test results of 23 biomass materials conducted by the Energy Technology Laboratory, part of the Energy and Environmental Research Division at the Thailand Institute of Scientific and Technological Research *[*30], this study evaluates 23 types of biomasses, treating each as a DMU. The resulting decision matrix is presented in Table 1.

**Table 1:** The decision matrix for evaluating the potential of 23 biomass materials.

DMUs (Alternatives)	*x_1_* (*C_1_*)	*x_2_* (*C_2_*)	*x_3_*(*C_3_*)	*y_1_*(*C_4_*)	*y_2_*(*C_5_*)
Cotton Stalks (DMU1)	9.33	4.77	67.95	17.95	4044.29
Sorghum (DMU2)	4.31	8.63	68.83	18.23	4051.48
Corn Stalks (DMU3)	13.32	6.20	64.58	15.9	4313.90
Corn Cobs (DMU4)	4.39	1.03	80.17	14.41	4187.00
Coconut Shells (DMU5)	11.79	0.85	64.03	23.33	4860.48
Palm Shells (DMU6)	13	1.30	64.55	21.05	5072.50
Rice Straw (DMU7)	2.86	11.24	65.64	20.26	3503.51
Durian Peel (DMU8)	9.93	2.71	74.3	13.06	4449.45
Sunflower Residue (DMU9)	11.5	3.67	64.34	20.49	4034.20
Bagasse (DMU10)	13.38	2.61	64.73	19.26	3972.76
Rain Tree Leaves (DMU11)	7.32	15.65	62.35	14.68	5078.74
Rice Husk (DMU12)	7.27	14.07	60.87	17.79	4009.40
Cassava Stalks (DMU13)	31.54	6.22	47.73	14.51	4670.00
Cassava Rhizomes (DMU14)	41.98	3.57	41.86	12.59	4368.30
Signal Grass (DMU15)	5.91	8.04	66.97	19.08	3939.68
Cogon Grass (DMU16)	5.75	6.53	65.32	22.4	3773.11
Tribulus (DMU17)	8.57	9.88	65.23	16.32	4340.92
Giant Sensitive Plant (DMU18)	9.25	4.15	64.38	22.22	4556.10
Water Hyacinth (DMU19)	6.47	10.08	67.07	15.7	3492.13
Rubberwood (DMU20)	3.94	4.54	16	73.52	6934.02
Eucalyptus wood (DMU21)	4.3	1.51	79.1	15.09	4436.00
Sawdust from deciduous trees (DMU22)	7.87	2.23	72.14	17.76	5179.00
Giant mimosa wood (DMU23)	9.09	1.03	72.17	17.71	4309.40

After obtaining the decision matrix for evaluating the potential of 23 biomass materials, the criteria weights were calculated using the BWM.

The initial step of BWM involves identifying the most and least favorable selection criteria. In this context, " calorific value (*y_3_* or *C_5_*)" is considered the most favorable criterion, while " volatile matter (*x_3_* or *C_3_*)" is regarded as the least desirable.

In the second step, the preferences of the best criterion compared to the other criteria are determined by calculating the average scores from the input provided by five decision-makers, as shown in Table 2.

**Table 2:** The BO vector.

Criteria Best criterion: *C_5_*	*x_1_* (*C_1_*)	*x_2_* (*C_2_*)	*y_1_*(*C_3_*)	*y_2_*(*C_4_*)	*y_3_*(*C_5_*)
Expert 1	4.0	2.0	4.0	1.0	1.0
Expert 2	3.0	2.0	4.0	2.0	1.0
Expert 3	4.0	1.0	3.0	2.0	1.0
Expert 4	3.0	2.0	4.0	2.0	1.0
Expert 5	4.0	2.0	3.0	1.0	1.0
Geometric mean	3.57	1.74	3.57	1.52	1.00

In the third step, the preferences of the criteria relative to the least important factor are determined by calculating the average score based on the input of five decision makers, as shown in Table 3.

**Table 3:** The OW vector.

Criteria Worst criterion: *C_3_*	*x_1_* (*C_1_*)	*x_2_* (*C_2_*)	*y_1_*(*C_3_*)	*y_2_*(*C_4_*)	*y_3_*(*C_5_*)
Expert 1	1	2	1	3	4
Expert 2	1	2	1	2	3
Expert 3	1	2	1	3	4
Expert 4	2	2	1	2	3
Expert 5	1	2	1	3	4
Geometric mean	1.15	2.00	1.00	2.55	3.57

**Fig. 2:**
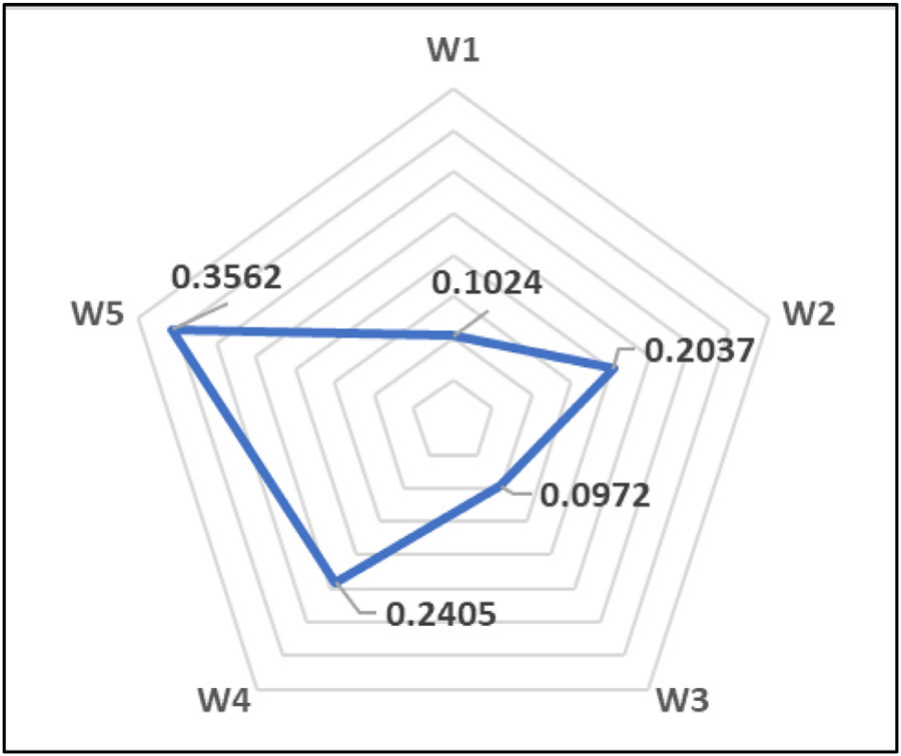
The Spearman correlation coefficients for evaluating the potential of 23 biomass materials.

Table 4 presents the Lingo code based on the BWM used to determine the criteria weights for evaluating the potential of 23 biomass materials. By analyzing the data from Table 2, Table 3 and applying BWM Eqs. (2) through (6), the resulting weights are illustrated in Fig. 2.

**Table 4:** The details of the Lingo code based on BWM for obtaining the criteria weights.

As seen in Fig. 2 the criteria weights derived as follows: *w*_1_* = 0.1024, *w*_2_* = 0.2037, *w*_3_* = 0.0972, *w*_4_* = 0.2405, and *w*_5_* = 0.3562. Furthermore, the consistency ratio ($\varepsilon^{L*}$) is calculated to be 0.0093, indicating a high level of reliability. The results of this numerical case demonstrate the ease and consistency of the BWM method. After obtaining the criteria weights, using the dataset presented in Table 1 (decision matrix), the efficiency scores ($\theta_{k}$) were calculated employing the DEAV model, as outlined in Eqs. (10) to (13). For example, the efficiency of DMU1 can be determined as below.

$$\begin{array}{ccc} Max\;\;\theta_{1} & = & \left(17.95^{0.2405}\right)u_{1}+\left(4044.29^{0.3562}\right)u_{2}\\ & & \left(9.33^{0.1024}\right)v_{1}+\left(4.77^{0.2037}\right)v_{2}+\left(67.95^{0.0972}\right)v_{3}=1,\\ & & \left(\left(17.95^{0.2405}\right)u_{1}+\left(4044.29^{0.3562}\right)u_{2}\right)-\left(\left(9.33^{0.1024}\right)v_{1}+\left(4.77^{0.2037}\right)v_{2}+\left(67.95^{0.0972}\right)v_{3}\right)\leq 0,\\ & & \left(\left(18.23^{0.2405}\right)u_{1}+\left(4051.48^{0.3562}\right)u_{2}\right)-\left(\left(4.31^{0.1024}\right)v_{1}+\left(8.63^{0.2037}\right)v_{2}+\left(68.83^{0.0972}\right)v_{3}\right)\leq 0,\\ & & \left(\left(15.90^{0.2405}\right)u_{1}+\left(4313.90^{0.3562}\right)u_{2}\right)-\left(\left(13.32^{0.1024}\right)v_{1}+\left(6.20^{0.2037}\right)v_{2}+\left(64.58^{0.0972}\right)v_{3}\right)\leq 0,\\ & & \vdots \\ & & \left(\left(17.71^{0.2405}\right)u_{2}+\left(4309.40^{0.3562}\right)u_{3}\right)-\left(\left(9.09^{0.1024}\right)v_{1}+\left(1.03^{0.2037}\right)v_{2}+\left(72.17^{0.0972}\right)v_{3}\right)\leq 0,\\ & & u_{1},u_{2},v_{1},v_{2},v_{3}\geq 0. \end{array}$$

This linear programming model can be solved using various optimization software. However, in this study, Lingo software was utilized to compute all the efficiency scores for this problem, as detailed in Table 5.

**Table 5:** The details of the Lingo code based on the DEAV model for the potential evaluation of 23 biomass materials.

After running the Lingo code, the $\theta_{k}$values are displayed in Table 6.

**Table 6:** The ranking of 23 biomass materials.

DMUs (Alternatives)	$\theta_{k}$	Rank
Cotton Stalks (DMU1)	0.7507	13
Sorghum (DMU2)	0.7367	17
Corn Stalks (DMU3)	0.7439	16
Corn Cobs (DMU4)	0.8449	5
Coconut Shells (DMU5)	0.8837	2
Palm Shells (DMU6)	0.8704	3
Rice Straw (DMU7)	0.6997	21
Durian Peel (DMU8)	0.7937	8
Sunflower Residue (DMU9)	0.7620	11
Bagasse (DMU10)	0.7697	10
Rain Tree Leaves (DMU11)	0.7449	15
Rice Husk (DMU12)	0.6993	22
Cassava Stalks (DMU13)	0.7482	14
Cassava Rhizomes (DMU14)	0.7528	12
Signal Grass (DMU15)	0.7288	20
Cogon Grass (DMU16)	0.7337	18
Tribulus (DMU17)	0.7307	19
Giant Sensitive Plant (DMU18)	0.7930	9
Water Hyacinth (DMU19)	0.6844	23
Rubberwood (DMU20)	1.0000	1
Eucalyptus wood (DMU21)	0.8442	6
Sawdust from deciduous trees (DMU22)	0.8567	4
Giant mimosa wood (DMU23)	0.8398	7

As seen in Table 6, the ranking of 23 biomass materials is based on their performance metric $\theta_{k}$​. Rubberwood (DMU20) achieves the highest score of 1.0000, securing the top rank, while Water Hyacinth (DMU19) has the lowest score of 0.6844, placing it at the bottom of the list. Notable high performers include Coconut Shells (DMU5) and Palm Shells (DMU6), with scores of 0.8837 and 0.8704, ranking them 2nd and 3rd, respectively. Corn Cobs (DMU4) also rank prominently at 5th with a $\theta_{k}$value of 0.8449. The data highlights the performance variations among the biomass materials, facilitating informed decisions for their selection based on their efficiency and suitability for specific applications.

### Method validation

In this section, a comparative analysis was conducted using various MADM techniques. This analysis utilized data obtained from the DEAV model, employing the decision matrix from Table 1 with weights assigned as *w*_1_* = 0.1024, *w*_2_* = 0.2037, *w*_3_* = 0.0972, *w*_4_* = 0.2405, and *w*_5_* = 0.3562. Table 7 provides a detailed comparison of the proposed method with other MADM approaches, including TOPSIS [11], WASPAS [12], COPRAS [14], and MOORA [15], and GRA [16].

**Table 7:** The ranking comparison of the proposed DEAV model against established MADM methods.

DMUs	DEAV	Rank	TOPSIS	Rank	WASPAS	Rank
1	0.7507	13	0.3893	13	0.3378	15
2	0.7367	17	0.3497	16	0.3424	13
3	0.7439	16	0.3510	15	0.3192	19
4	0.8449	5	0.4382	7	0.4804	4
5	0.8837	2	0.4795	2	0.5421	2
6	0.8704	3	0.4634	3	0.4817	3
7	0.6997	21	0.3236	18	0.3420	14
8	0.7937	8	0.4043	11	0.3615	10
9	0.7620	11	0.4120	10	0.3560	11
10	0.7697	10	0.4162	9	0.3659	9
11	0.7449	15	0.2757	22	0.3260	17
12	0.6993	22	0.2714	23	0.3029	22
13	0.7482	14	0.3057	20	0.3164	20
14	0.7528	12	0.3316	17	0.3224	18
15	0.7288	20	0.3543	14	0.3306	16
16	0.7337	18	0.3906	12	0.3463	12
17	0.7307	19	0.3127	19	0.3153	21
18	0.7930	9	0.4291	8	0.3832	8
19	0.6844	23	0.3045	21	0.2888	23
20	1.0000	1	0.8838	1	0.7471	1
21	0.8442	6	0.4391	6	0.4527	6
22	0.8567	4	0.4476	4	0.4352	7
23	0.8398	7	0.4446	5	0.4717	5
1	0.4491	13	0.0412	13	0.4653	12
2	0.4137	15	0.0262	16	0.4500	15
3	0.4126	16	0.0275	15	0.4480	16
4	0.5853	3	0.0624	8	0.5452	5
5	0.6069	2	0.0874	2	0.5653	2
6	0.5722	5	0.0813	3	0.5576	3
7	0.3830	18	0.0090	18	0.4318	21
8	0.4722	11	0.0470	11	0.4988	8
9	0.4766	10	0.0509	9	0.4777	11
10	0.4790	9	0.0506	10	0.4871	10
11	0.3776	20	−0.0094	22	0.4445	18
12	0.3621	21	−0.0104	23	0.4141	23
13	0.3803	19	0.0073	19	0.4441	19
14	0.3579	22	−0.0003	21	0.4601	13
15	0.4171	14	0.0278	14	0.4457	17
16	0.4526	12	0.0421	12	0.4581	14
17	0.3915	17	0.0139	17	0.4350	20
18	0.5097	8	0.0639	7	0.4960	9
19	0.3572	23	−0.0001	20	0.4163	22
20	1.0000	1	0.2347	1	0.9269	1
21	0.5760	4	0.0656	6	0.5429	6
22	0.5618	6	0.0760	4	0.5499	4
23	0.5614	7	0.0667	5	0.5379	7

Table 7 shows the efficiency scores and the corresponding ranks for 23 biomass materials (DMUs) using various methods. The results indicate that Rubberwood (DMU20) consistently achieves the highest rank (1st) across most methods, with a perfect DEAV score of 1.0000, indicating its superior suitability for charcoal briquette production. On the other hand, Water Hyacinth (DMU19) ranks lowest in the DEAV model with a score of 0.6844 and consistently performs poorly across other methods, highlighting its inadequacy as a biomass material. Notable top performers include Coconut Shells (DMU5) and Palm Shells (DMU6), which are ranked highly across all methods, demonstrating their high potential for charcoal briquette production.

**Fig. 3:**
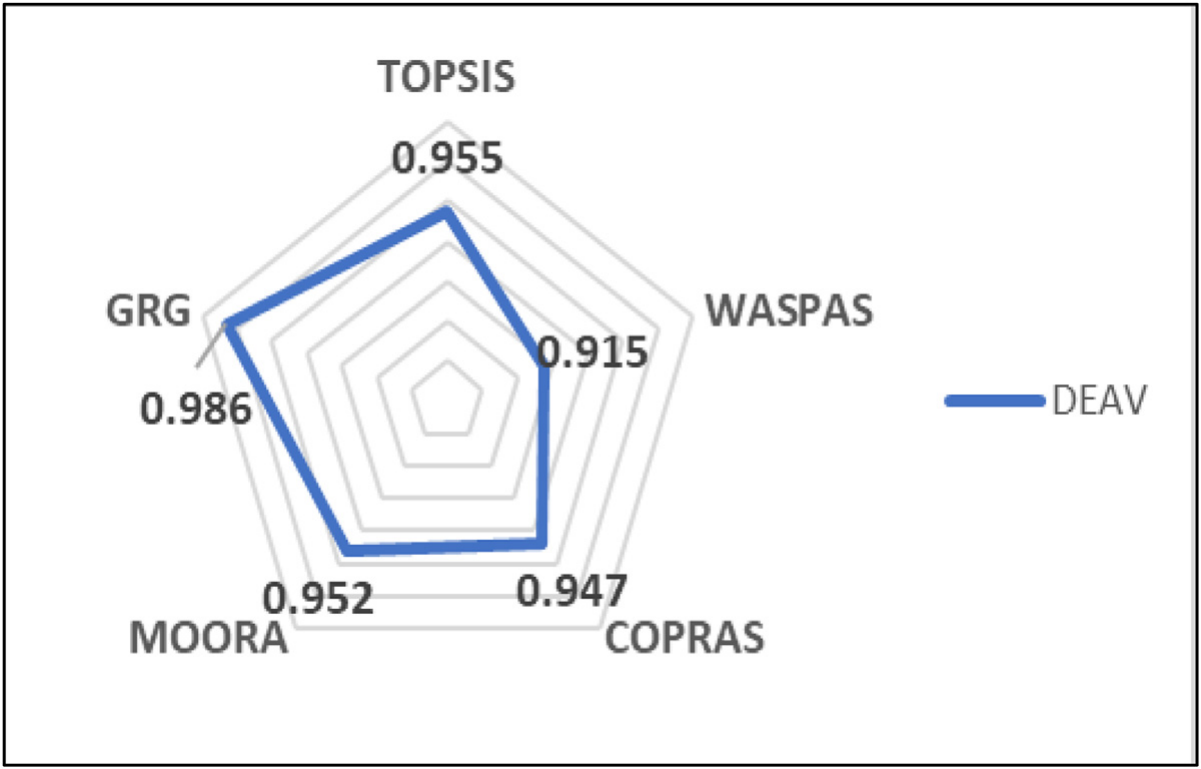
The Spearman correlation coefficients for the proposed method and other MADM methods.

In addition, Fig. 3 shows the Spearman correlation coefficients for the comparison between the DEAV model and other MADM methods.

Fig. 3 illustrates the Spearman correlation coefficients between the proposed method (DEAV) and various other MADM methods, including TOPSIS, WASPAS, COPRAS, MOORA, and GRG. The correlation coefficients provide a measure of the rank correlation between the methods. The proposed DEAV method exhibits the highest correlation with GRG (0.986), indicating a strong agreement in rankings between these two methods. The correlation with TOPSIS is also high (0.955), followed by MOORA (0.952), COPRAS (0.947), and WASPAS (0.915). This figure highlights the consistency of the proposed method with established MADM approaches, demonstrating its robustness and reliability in ranking biomass materials.

## Conclusions and limitations

The sustainable conversion of agricultural waste into charcoal briquettes is a significant advancement in addressing energy shortages and waste management, especially in agricultural nations like Thailand. This study introduces a novel methodology that integrates the Data Envelopment Analysis Variant (DEAV) with the Best-Worst Method (BWM) to provide a robust framework for evaluating and ranking biomass materials for charcoal briquette production. By addressing multiple attribute decision-making (MADM) challenges, this methodology enhances the decision-making process by incorporating both efficiency and consensus among various criteria. Empirical testing on 23 different biomass materials demonstrated the method's effectiveness, with Rubberwood (DMU20) achieving the highest performance score and Water Hyacinth (DMU19) the lowest. The Spearman correlation coefficients indicated a strong agreement between the DEAV method and other MADM methods, with the highest correlation observed with Grey Relational Analysis (GRG) (0.986), followed by TOPSIS (0.955), MOORA (0.952), COPRAS (0.947), and WASPAS (0.915). The DEAV-BWM model's comprehensive approach ensures a more accurate and practical evaluation, making it valuable for researchers and practitioners in sustainable energy, industrial engineering, and decision science.

Key contributions of this study include advancing MADM methodology, validating DEAV through strong correlations with established methods, and providing a versatile evaluation framework adaptable to various contexts. Practically, the model offers an effective tool for identifying suitable biomass materials, enhancing decision-making, and presenting a methodology that is adaptable for diverse applications. This underscores the significance of the DEAV-BWM model for future research and practical implementations in sustainable energy.

Despite the promising results, the proposed DEAV-BWM method has some limitations. First, the reliance on expert judgment for criteria weighting can introduce subjective biases, potentially affecting the reliability of the results. Efforts to standardize and validate these weights across different contexts and applications are necessary to enhance the robustness of the model. Second, the current validation of the method is limited to biomass materials for charcoal briquette production, which may restrict its generalizability to other types of biomass or alternative energy applications. Third, while the DEAV-BWM method is comprehensive, it may be computationally intensive, particularly when applied to larger datasets or more complex decision-making scenarios. Based on the provided content and focus of the research, here are the Future Research Directions for the study:

1. **Expand Empirical Testing:** The current validation is focused on biomass for charcoal briquette production. Expanding empirical testing to include a wider variety of biomass materials and alternative energy sources would validate the robustness and versatility of the proposed method.
2. **Expand Applications and Foster Cross-Disciplinary Collaborations:** The DEAV-BWM framework, while primarily focused on charcoal briquette production, holds significant potential for broader applications in sustainability-related sectors. Future studies could explore its adaptability in areas such as biofuel production, environmental impact assessments, and sustainable product development, showcasing its versatility across diverse decision-making scenarios. Additionally, engaging in cross-disciplinary collaborations with experts from fields like environmental science, industrial engineering, and operations research would provide diverse perspectives, enrich the decision-making process, and enhance the practical relevance of the method.
3. **Standardize Criteria Weight Determination:** The reliance on expert judgment for criteria weighting introduces subjectivity. Future research should focus on developing standardized approaches for determining weights, potentially integrating statistical methods like the Analytic Hierarchy Process (AHP) or Delphi Method to enhance objectivity and reliability.
4. **Incorporate Advanced Decision-Making Techniques:** To improve the robustness of the evaluation process, integrating advanced decision-making techniques such as **Fuzzy Logic** could offer more effective handling of uncertainty and complexity.
5. **Conduct Sensitivity and Robustness Analyses:** Additional sensitivity analyses are recommended to examine the impact of varying criteria weights and input data on final rankings. These analyses would enhance the stability and reliability of the DEAV-BWM model, making it a more dependable decision-making tool.
6. **Develop User-Friendly Decision-Support Tools:** To facilitate broader adoption, creating user-friendly software tools or decision-support systems that automate data input, criteria weighting, and analysis would make the DEAV-BWM method accessible to a wider audience, including non-experts

## Declaration of competing interest

The authors declare that they have no known competing financial interests or personal relationships that could have appeared to influence the work reported in this paper.

## Data Availability

Data will be made available on request.
